# Arthroscopy provides symptom relief and good functional outcomes in patients with hip synovial chondromatosis

**DOI:** 10.1093/jhps/hnv044

**Published:** 2015-07-13

**Authors:** Fernando P. Ferro, Marc J. Philippon

**Affiliations:** Steadman Philippon Research Institute, Vail, CO 81657, USA

## Abstract

The purpose of this study was to evaluate clinical presentation, associated pathology and clinical outcomes after arthroscopic treatment of hip synovial chondromatosis (SC). A prospective data registry was queried for patients with SC diagnosis from 2005 to 2012. Surgical indications were intra-articular pain after failure of conservative treatment, labral pathology, chondral damage or loose bodies. All patients had femoroacetabular impingement based on radiographic findings. Patient-centered outcomes were collected before and after surgery. Standard hip arthroscopy techniques were used to address associated pathology. Twenty-three patients met the inclusion criteria. Eleven were males. Mean age was 43.7 years. Mean center-edge angle was 33.7 and alpha angle 73. Radiographs were diagnostic in five patients (23.8%). Magnetic resonance imaging identified loose bodies in 14 (66%). Most patients had an uncountable amount of loose bodies in the central and peripheral compartments. The most common associated pathology was a labral tear (100%) and acetabular cartilage injury (85%). All patients had improvement in range of motion. The average Modified Harris Hip score improved from 62 (pre-op) to 84.8 (post-op). Short-Form 12-PCS improved from 41 to 53. Western Ontario and McMaster Osteoarthritis Index improved from 27.1 to 7.2. Median overall satisfaction was 9.5 (out of 10). Hip arthroscopy with thorough removal of loose bodies and subtotal synovectomy, coupled with an aggressive and early rehabilitation program, was effective in ameliorating symptoms associated with from hip SC, yielding high levels of patient satisfaction and functional outcomes, in a 2.5 year follow-up time. Level of evidence: IV (case series)

## INTRODUCTION

Synovial chondromatosis (SC) is a rare benign proliferative disorder where multiple metaplastic cartilaginous masses form within the synovial membrane [1]. Histological analysis shows metaplasia of synovial mesenchymal cells [2]. They may even calcify or ossify with time [3]. Most often the presentation is mono-articular and involves the knee, the hip being second most affected [3]. In rare instances, it might be proliferative and cause extensive bone erosions, subluxation and secondary joint degeneration [4, 5].

The exact etiology is not yet completely understood, although it has been identified decades ago [3]. Clinical presentation usually is a dull joint pain, catching, limitation of range of motion (ROM) or snapping. It might be diagnosed with radiographs (joint space widening), but the magnetic resonance imaging (MRI) will often yield a higher sensitivity. This entity is probably under-diagnosed for lack of suspicion, since initial radiographs are frequently normal [6].

Open hip surgery with joint dislocation has been an accepted treatment option; however, the recovery periods have been shown to be long, with a high risk of complications [7]. Treatment may warrant subtotal synovectomy, but masses can still recur. There is also the associated risk of osteonecrosis of the femoral head when an open arthrotomy is done, especially if a meticulous surgical dislocation technique is not followed during exposure of the peripheral compartment distally and posteriorly. Other complications include neurovascular injury, deep vein thrombosis and wound infection [7].

Recently, different reports have described the arthroscopic approach to this condition with good results [1, 2, 8, 9]. Although a truly total synovectomy is not possible with this technique, it is possible to remove loose bodies, improve symptoms and possibly delay the development of arthritis. Besides, other intra-articular and synovial conditions have been successfully treated through minimally invasive strategies, such as pigmented villonodular synovitis and inflammatory arthropathies [10, 11]. The purpose of this study was to determine the outcomes and recurrence rates in a consecutive series of patients undergoing hip arthroscopy by a single surgeon for the treatment of SC, associated with femoroacetabular impingement (FAI). We hypothesized that hip arthroscopy is an effective treatment for this rare condition.

## METHODS

A prospective data registry was queried to identify all patients that underwent surgery between March 2005 and June 2012 and had a confirmed diagnosis of SC after anatomic pathology analysis. All patients were treated at a referral center for hip arthroscopy. All patients presented with disabling hip pain and prospectively enrolled in an Internal Review Board (IRB) approved study.

Surgical indications included signs of intra-articular pain, nonresponsive to conservative management of at least 3 months and MRI diagnosis of labral pathology, chondral damage or loose bodies. The primary indication for surgery was not necessarily SC, since many patients did not have this diagnosis preoperatively. For patients who were not diagnosed with SC before surgery, the main surgical indication was FAI. Patients were then included in the study if a diagnosis of SC was confirmed by biopsy and typical arthroscopic findings. Patients were excluded if they were less than 18 years of age, did not have SC or lacked follow-up information.

Before surgery, all patients were evaluated with plain radiographs. Measurements were done for center edge angle (AP view) and alpha angle (cross-table lateral view). MRI was done for every case. The presence of loose bodies at MRI was documented. ROM (flexion, abduction, adduction, external and internal rotation) was assessed both pre- and post-operatively. Patients completed subjective forms preoperatively and at follow-up, personally, through mail or e-mail. The forms were then uploaded to a custom database software that backs up all information and allows for score calculation. Multiple outcome scores were calculated from this data: the Modified Harris Hip Score (mHHS) [12], Short-Form 12 (SF-12) [13] and the Western Ontario and McMaster Osteoarthritis Index (WOMAC) [14]. The mHHS [12] and WOMAC [14] are subjective scores that include questions about pain, limp, use of support, walking distance and functional activities. The SF-12 [13] is a general health score that includes questions about general health, limitation for daily activities, physical and emotional health. Patient satisfaction with outcome was also collected (zero being totally unsatisfied, 10 being totally satisfied).

All patients underwent a hip arthroscopic surgical procedure by a single surgeon, using the supine position on a traction table and two working portals (anterolateral and mid-anterior). An inter-portal capsulotomy was routinely done. A thorough joint inspection was carried out including the entirety of the central and peripheral compartments. Arthroscope and instruments were switched between portals multiple times to allow ample access to the whole joint for identification and removal of all loose bodies. Traction was released and the hip was moved into about 70° of flexion and various degrees of rotation for a complete evaluation of the peripheral compartment. Full flexion and external rotation allowed for anterior capsule relaxation and better access to the most medial portions of the peripheral compartment. Extension coupled with internal rotation allowed better access to the most lateral aspects of the compartment. By having a dedicated assistant moving the leg through various positions, we could effectively expose and clean the entirety of the peripheral compartment.

Cam and pincer lesions were removed according to pre-op radiological evaluation and intra-op dynamic assessment of FAI. Labral tears were repaired if necessary [15]. Cartilage lesions were treated with thermal chondroplasty and/or microfracture if they were full-thickness [16]. Loose bodies were manually removed using an arthroscopic shaver and grasper, or washed away until a totally clean joint was achieved. A thorough arthroscopic synovectomy using a shaver and radiofrequency device was carried out when SC was suspected after arthroscopic inspection. Intra-operative findings were prospectively documented in a standardized research sheet.

After surgery, patients were kept in partial weight bearing for 3 weeks. Stationary bike without resistance was employed from first day after surgery. A non-steroidal anti-inflammatory drug was used to prevent heterotopic ossification and pain control. In case of persistent articular pain after 6 months of follow-up, a postoperative MRI was done to investigate disease recurrence.

### Statistical analysis

For comparison of continuous variables, the Pearson’s correlation coefficient was used. For comparison of continuous variables the independent *t*-test was used for two variable and one-way ANOVA was used for more than two variables. Comparison between preoperative scores and postoperative scores was done with a paired *t*-test. For patient satisfaction, nonparametric univariate analysis was performed. The Spearman’s rho correlation coefficient (*r*) was used for assessing associations between continuous nonparametric variables.

## RESULTS

Twenty-three patients met the inclusion criteria. Preoperative information is shown in Table I. There were 11 were males and 12 females. Mean age at surgery was 43.7 years (range 24–58).

**Table I.** Patient demographics and radiologic assessment

	Mean (SD)
Age	43.7 (9.9) years
Gender	11 males, 12 females
Center-edge angle	33.7 (7.8)°
Alpha angle	73 (9.6)° (all alpha angles were 55° or greater)
Loose bodies visible on radiographs	5 (23.8%)
Loose bodies visible on MRI	14 (66.6%)
Tonnis grade	Grade 0 in 14 patients (66.6%)
	Grade 1 in 7 patients (28.5%)
	Grade 2 in one (4%)

**Fig. 1.**
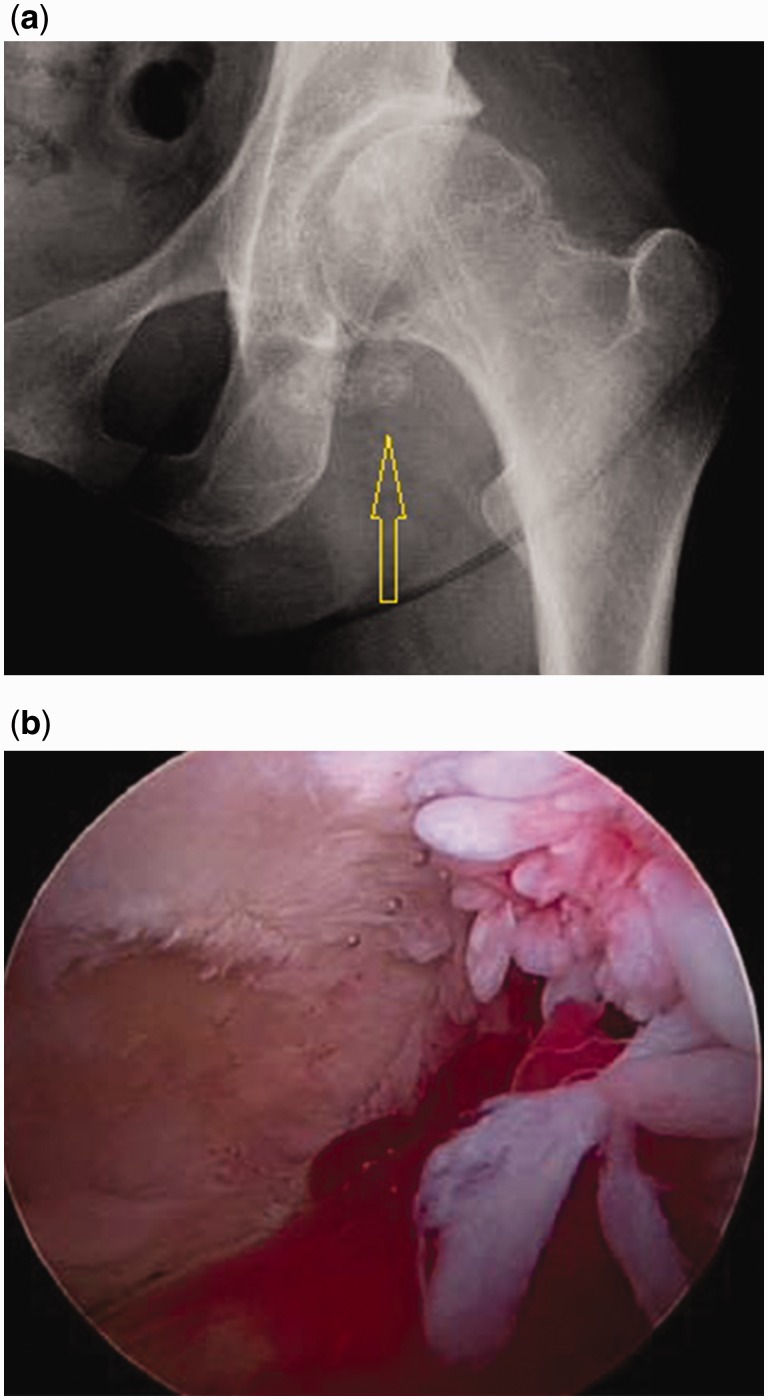
(**a**) Anteroposterior hip radiograph with visible loose bodies(arrow). (**b**) Intraoperative photo showing inflamed synovium and degenerative changes in the joint.

Radiographs were seldom able to suggest the diagnosis of SC, because of visible calcified loose bodies in the joint space. That happened in only five patients (23.8%) (Fig. 1).

**Fig. 2.**
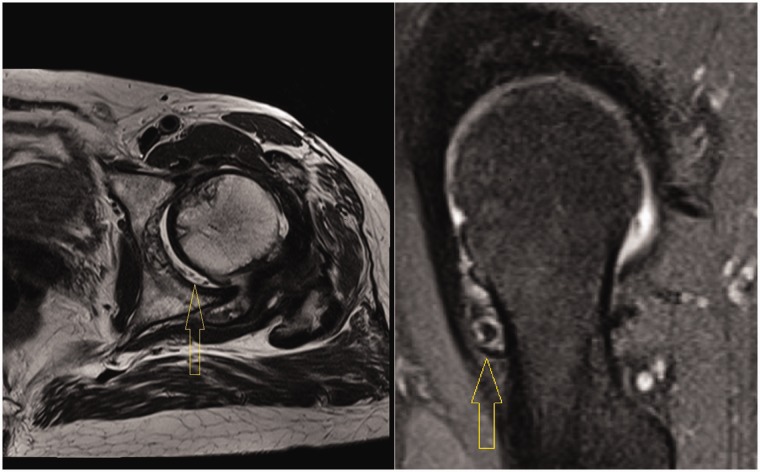
MRI showing loose bodies in the joint space, in two different cases.

**Fig. 3.**
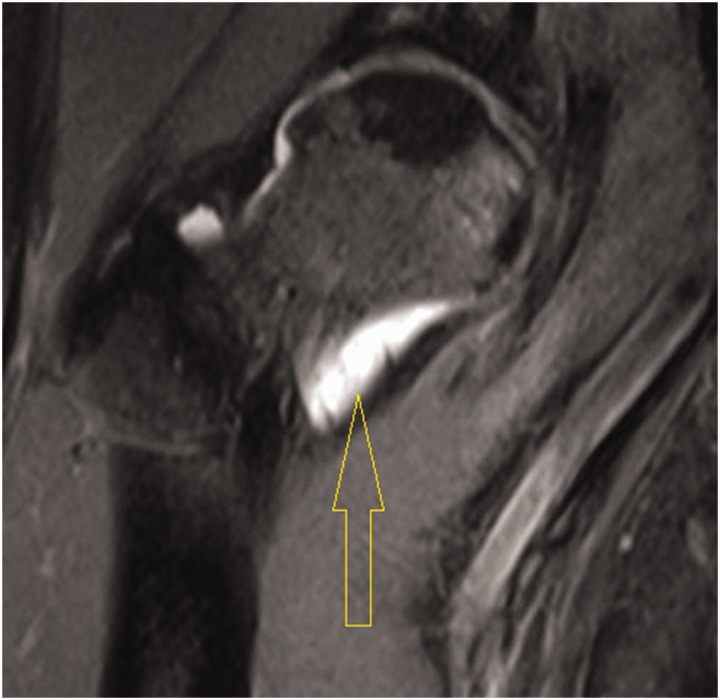
MRI showing significant joint effusion.

MRI detected loose bodies in 14 patients (66.6%) (Fig. 2). MRI was not able to detect the loose bodies in any of the seven patients with small amounts of loose bodies. It did, however, show evidence of joint effusion and synovial thickening in all patients (Fig. 3).

**Fig. 4.**
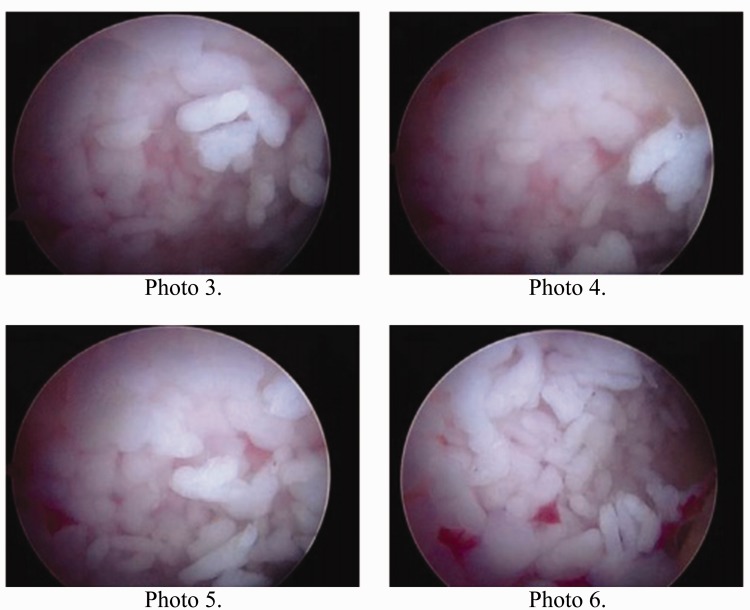
Loose bodies inside the hip joint as seen at hip arthroscopy.

**Fig. 5.**
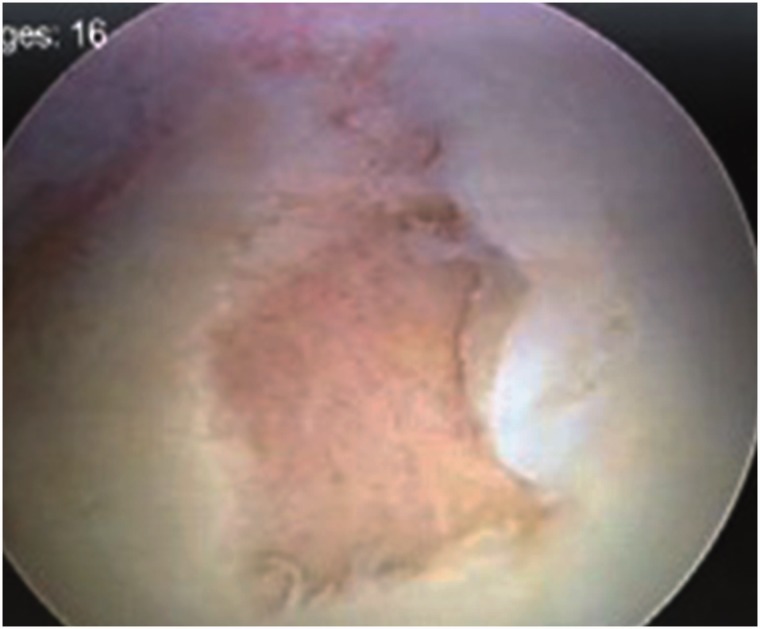
Acetabular chondral lesion identified at arthroscopy.

Most patients in this study had too many loose bodies to count (Fig. 4). Seven patients had a countable amount of loose bodies, averaging 11.57 bodies. They were variable in size and shape, often aggregated as clumps. They were frequently concentrated in the cotyloid fossa and capsular recesses, and all patients had loose bodies in both the central and peripheral compartments. In addition to extensive synovitis and multiple loose bodies, which are the hallmark of SC, most patients had some form of associated intra-articular pathology, such as labral tears, ligamentum teres tears, acetabular or femoral chondral defects. The incidence of such associated pathology is summarized in Table II. The most common pathology was labral tears followed by acetabular cartilage injury (Fig. 5). The high incidence of labral tears and the high average alpha angle (73°) suggest a frequent association between cam-type femoroacetabular impingement and SC. This can be seen on Figs. 1–3, which all have bony deformities at the femoral neck junction.

**Table II.** Associated hip pathologies and treatments identified at hip arthroscopy for patients with SC

	Number of patients	Percentage
Ligamentum teres tears	11	52
Chondral lesion—femur	12	57
Chondral lesion—acetabulum	18	86
Labral pathology	21	100
Capsular adhesions	2	10

Pre- and post-operative ROM (at 3 months post-op) is summarized in Table III. Subjective scores were assessed before and after surgery, at an average of 30.0 months (range 15–68) (Table IV).

**Table III.** Preoperative and postoperative ROM (degrees) in patients treated for SC

	Pre-operatively, mean (SD)	Follow-up, mean (SD)	*P*-value
Flexion	108 (14)	116 (11)	0.001
Abduction	44.6 (14)	47 (10)	0.877
Adduction	20.6 (11)	25 (10)	0.728
Internal rotation	23 (13)	27.5 (7)	0.032
External rotation	38.5 (16)	41.1 (9)	0.723

**Table IV.** Outcome scores for patient treated with hip arthroscopy for SC

	Pre-operatively, mean (SD)	Follow-up, mean (SD)	*P*-value
MHHS	62 (15)	84.8 (12)	0.003
SF12 MCS	56 (10)	55.8 (5)	0.176
SF12 PCS	41.6 (8)	53 (8)	0.009
WOMAC	27.1 (17)	7.2 (7)	0.004
**Median overall satisfaction**	**9.5**		

SF12MCS/PCS, short form-12 physical component summary/mental component summary.

In all patients with arthroscopic findings that were consistent with the diagnosis of SC, a biopsy was performed. Pathology reports showed chronic synovitis, osteocartilaginous nodules with hyaline cartilage, and areas of irregular and patchy calcification within the synovial membrane.

No cases of major complications such as infection, thromboembolism or severe persistent pain were reported. We report no cases of the rare complication of chondrosarcoma.

Two patients required a total hip replacement. One patient who required a total hip replacement was a 50-year-old female. Thirteen months following her initial arthroscopy, a revision arthroscopy was performed. We again observed extensive loose bodies and a grade 4 chondral lesion on the femoral head. Two years following the revision, the third arthroscopy was done, in which we observed disease recurrence, extensive adhesions and worsening of the chondral defect, for which we attempted a microfracture procedure. Despite this, the patient needed a total hip replacement 4 years after the initial hip arthroscopy. The other patient who required a total hip replacement was a 49-year-old female. She underwent only one arthroscopic procedure. During surgery, several loose bodies were removed. There were grade 4 acetabular chondral lesions that required microfracture. During follow up, she underwent a total hip replacement after progression to arthritis. The other 21 of the 23 patients did not require a second surgery.

## DISCUSSION

SC is one of several diseases that, without early diagnosis and treatment, would later cause ‘idiopathic osteoarthritis.’ With the widespread recognition of hip disease etiologies such as FAI, osteonecrosis and auto-immune diseases, we believe the number of ‘idiopathic’ degenerative hip disease will steadily fall. This study showed that SC can be effectively treated using hip arthroscopy with most patients reporting return to function and high satisfaction with outcome.

Before the development of current arthroscopic techniques, open surgery was the only effective treatment for this pathology. Lim *et al*. [7] reported a series of 21 patients treated by open surgery. Although they obtained improvement in Harris Hip scores, they found a significant number of complications, such as osteonecrosis of the femoral head, lesser trochanter fracture and femoral nerve palsy. Besides, the recovery period after open surgery is longer and morbid.

Our findings are consistent with previous reports, with good and excellent improvement in most patients, with the advantage of quick recovery time due to the minimally invasive nature of the procedure [1, 8, 9]. In this study, the average age was 43 years, which is expected for a disease that is typically diagnosed between 30 and 50 years of age since the earliest studies [3]. There was no evident gender predilection, which is also a typical finding.

Boyer *et al*. [8] have reported their experience with hip SC. Thirty-eight percent of their patients required revision surgery for recurrence, either open or arthroscopic. This contrasts with our study, in which revision surgery was necessary in only two patients (8.6%). In their study, however, 68% of patients did not have the central compartment inspected. In our series, 100% of patients had loose bodies within the central portion of the joint, and therefore we believe that joint distraction and thorough systematic inspection is mandatory. Other studies reported recurrence rates between 9% and 24% [1, 2, 7, 9]. These findings challenge the notion that SC is a chronic recurrent disease that will inevitably lead to arthritis.

During early stages of SC, the loose bodies are still smaller and rarely calcified. After some years, they become larger, ossified and thus visible in plain radiographs. This makes it harder to make the correct diagnosis before damage is done to the articular cartilage. In other studies, radiographs were suggestive or diagnostic in up to 50% of cases [8]. In our study, that was true in for only 23%.

MRI was more sensitive than radiographs, effectively identifying loose bodies in 66% of cases. These cases underwent surgery because of associated femoroacetabular impingement symptoms, during which the diagnosis of SC was made. This highlights the importance of a thorough arthroscopic inspection before starting any procedure, no matter what the MRI report states.

In previous studies, some correlation has been shown between degree of radiological or intra-operative joint degeneration and progression to arthritis and total hip replacement [1, 2, 7, 17]. Our findings reproduce that, since our good outcomes might be correlated to that fact that most of our patients were Tonnis grade 0 or 1. Even so, it is reasonable to conclude that such patients deserve a close follow-up, since the risk of degenerative joint disease still exists. In an attempt to minimize the risk of progression of chondral lesions, we performed microfracture whenever indicated [16].

It is important to emphasize the common association of femoroacetabular impingement and SC. In this study, the average alpha angle was above normal (indicating cam impingement), and a labral tear was the most common associated articular pathology. This association has also been reported in a recent report of five patients who had concomitant FAI and SC and were treated by surgical hip dislocation [18]. Although unproven, we feel FAI is a separate disease entity for most patients. Many of the patients included in this study were not diagnosed with SC prior to arthroscopy. They had arthroscopy for FAI and SC was discovered intraoperatively. We suggest that every patient undergoing hip arthroscopy for FAI should have a synovial biopsy if multiple loose bodies and extensive synovitis are observed after inspection. In patients who present with recurrent SC with moderate/severe osteoarthritis, a total hip arthroplasty may be indicated.

Although other studies have previously reported on the arthroscopic treatment of SC [1, 6, 8], ours is the only one that gathered data prospectively. We were also able to calculate multiple outcome scores, as well as ROM and subjective patient satisfaction. In addition, we provide an assessment of frequent associated pathology, providing a broader perspective of this disease’s usual clinical presentation.

### Limitations

One of the limitations of our study is that we present a case series, lacking a control group. It would probably be unethical to compare arthroscopy versus conservative treatment for this disease, due to the obvious benefits of surgery. However, a prospective randomized trial comparing arthroscopy versus open surgery might be an option. Our data were gathered prospectively, which is one of the strengths of our study. However, data analysis was retrospective. Our average follow up time of 30 months is intermediary when compared with other similar studies. Maybe our low number of recurrences and total hip replacements is due to the fact that the evolution to osteoarthritis takes a longer time period. Also, it is possible that we had silent recurrences that were not diagnosed, because we did not perform a routine post-op MRI for asymptomatic patients.

## CONCLUSION

Hip arthroscopy with thorough removal of loose bodies and subtotal synovectomy, coupled with an aggressive and early rehabilitation program, is an effective way of ameliorating symptoms from hip SC, yielding high levels of patient satisfaction and functional outcomes in a 2.5 years follow-up time.

## CONFLICT OF INTEREST STATEMENT

FPF - MJP declares the following potential conflict of interest or source of funding: receives royalties from Smith & Nephew, Linvatec, Bledsoe, DonJoy, and Arthrosurface; is a paid consultant for Smith & Nephew and MIS;and owns stock or stock options in Smith & Nephew, Arthrosurface, HIPCO, and MIS. Research or institutional support has been received from Smith & Nephew, Siemens, Ossur, and Arthrex.
